# Pulmonary artery intimal sarcoma: a case report and literature review

**DOI:** 10.1002/rcr2.530

**Published:** 2020-02-05

**Authors:** Ding‐Yu Chang, Kun‐Chang Lin, Jun‐Yen Pan, Hung‐Wei Liu, Shu‐Hung Kuo, Lin Lee

**Affiliations:** ^1^ Division of Pulmonary Medicine Kaohsiung Veterans General Hospital Kaohsiung City Taiwan; ^2^ Department of Critical Care Medicine and Cardiovascular Center Kaohsiung Veterans General Hospital Kaohsiung City Taiwan; ^3^ Department of Pathology and Laboratory Medicine Kaohsiung Veterans General Hospital Kaohsiung City Taiwan

**Keywords:** Chronic pulmonary thromboembolism, pulmonary artery, pulmonary embolism, pulmonary hypertension, sarcoma

## Abstract

Pulmonary artery intimal sarcoma is a rare disorder arising from the intima of the pulmonary artery. Histopathology reveals that it is a tumour cell of mesenchymal origin. The signs and symptoms include chronic shortness of breath and other features of right ventricular failure, which mimic chronic pulmonary thromboembolism. The definitive diagnosis can rarely be made based on the symptoms and signs alone, and other investigations including echocardiography, computed tomography, magnetic resonance imaging (MRI), and positron emission tomography (PET) are often required. The gold standard for diagnosis is tissue biopsy. The mainstay for treatment is surgery, and complete surgical resection with endarterectomy provides survival benefit. According to recent evidences, however, multimodal treatment provides better survival outcomes than monotherapy such as surgery alone. Despite the newer upcoming treatment strategies, patients with pulmonary intimal sarcoma continue to have a poor prognosis. We present a case of pulmonary artery intimal sarcoma and review the literature associated with the disease.

## Graphical abstract

Pulmonary artery intimal sarcoma is a rare disorder arising from the intimal wall of the pulmonary artery. As the tumour decreases the lumen of the pulmonary artery, patients usually present with symptoms and signs of right ventricular failure, and it has often been misdiagnosed as pulmonary thromboembolism. In this report, we present the case of a patient in whom the diagnosis of pulmonary artery sarcoma was made promptly and surgery was performed soon after.

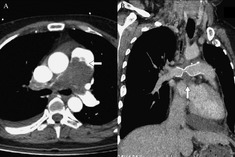

## Introduction

Pulmonary artery intimal sarcoma is a rare disorder arising from the intimal wall of the pulmonary artery. As the tumour decreases the lumen of the pulmonary artery, patients usually present with symptoms and signs of right ventricular failure, and it has been often misdiagnosed as pulmonary thromboembolism. It is important to differentiate between the two entities. In this report, we present the case of a patient in whom the diagnosis of pulmonary artery sarcoma was made promptly and surgery was performed soon after.

## Case Report

**Figure 1:**
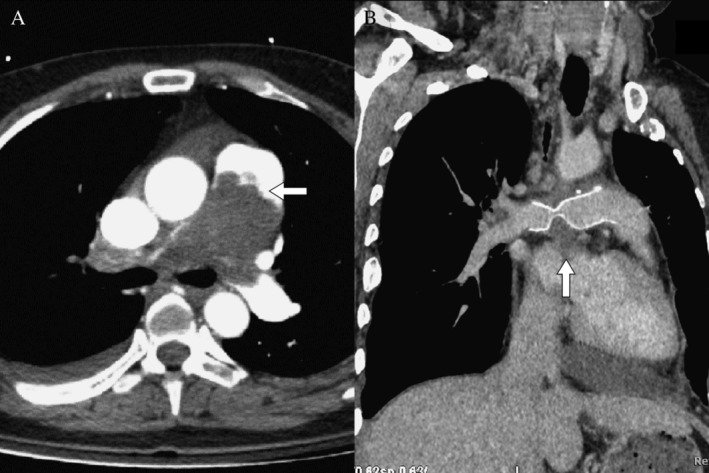
(A) Axial view of computed tomography (CT) angiography showing soft tissue‐like filling defects in the pulmonary trunk and extending into the left and right pulmonary arteries; the proximal edge of the mass was nodular structure (arrow) and the distal part of lesion was dilated and had a grape‐like appearance. There was heterogeneous contrast enhancement. (B) Chest computed tomography (CT) with contrast after operation showed residual tumour (arrow) with external compression on graft.

A 42‐year‐old woman presented with progressive shortness of breath for two months, accompanied by palpitation, pitting oedema, and fatigue. On the day of admission, she had an episode of presyncope. On physical examination, her vital signs showed a body temperature of 37°C, blood pressure of 157/103 mmHg, tachycardia with a heart rate of 126 bpm, and tachypnoea with a respiratory rate of 28 bmp. Her oxygen saturation (SpO_2_) was only 86% in room air. There was a visibly engorged jugular vein, palpable right ventricular heave, pansystolic murmur at the tricuspid area, fine crackles bilaterally over the lungs, and pitting oedema of the lower limbs bilaterally. The D‐dimer value was 1094.6 ng/mL (normal range < 500 ng/dL). Arterial blood gas values revealed respiratory alkalosis (pH: 7.46, partial pressure of carbon dioxide (pCO_2_): 30 mmHg). Complete blood count, liver function tests, and renal function tests were normal. Chest X‐ray showed cardiomegaly and bilateral pleural effusion. Electrocardiogram (EKG) revealed sinus tachycardia and right axial deviation. Echocardiography showed severe right ventricular systolic dysfunction, pulmonary artery hypertension, dilated right atrium, and dilated right ventricle. Considering the diagnosis of pulmonary thromboembolism, computed tomography (CT) was performed, which revealed nodular soft tissue‐like filling defects in the pulmonary trunk and right pulmonary arteries with heterogeneous contrast enhancement (Fig. 1). Pulmonary artery tumour was thus suspected; however, pulmonary embolism was not ruled out. Subsequent investigations revealed no risk of pulmonary embolism and no thrombus in the lower extremities on sonography, which favoured the diagnosis of pulmonary tumour.

The patient subsequently underwent surgical intervention with resection of the pulmonary artery mass for therapeutic management.

At surgery, frozen biopsy specimens obtained from the right main pulmonary artery revealed a pulmonary artery intimal sarcoma, and the patient then underwent resection of the tumour, resection of involved pulmonary artery, and reconstruction of the involved pulmonary artery with graft subsequently.

**Figure 2:**
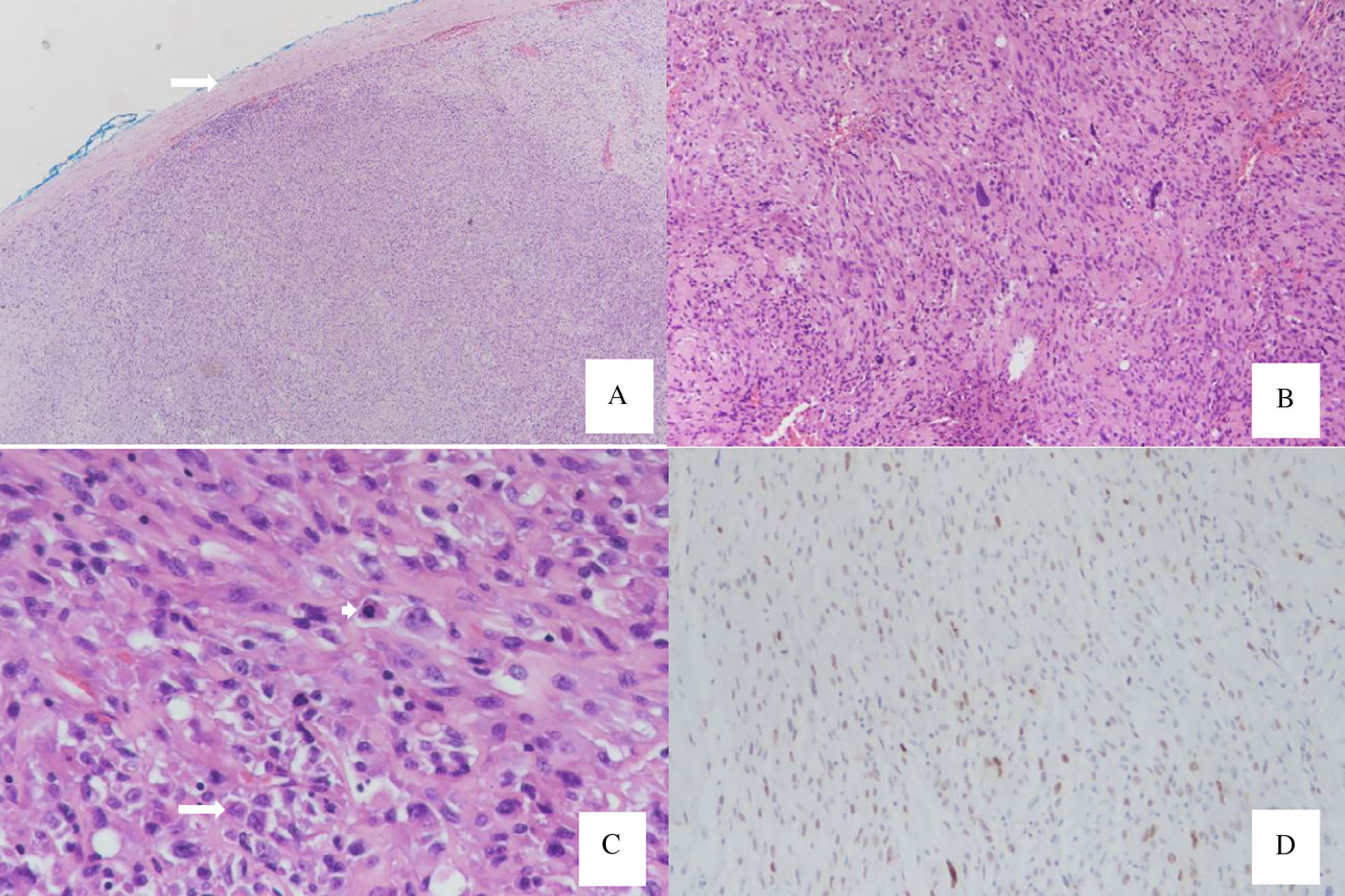
(A) 40× Haematoxylin and eosin (H&E) stain showed neoplastic cells in the vessel wall (arrow). (B) 100× H&E stain of neoplastic cell shows spindle growth arrangement. (C) 400× H&E stain of neoplastic cell shows spindle cells with variable cell pleomorphism (arrow) and mitosis (short arrow). (D) 400× Immunohistochemical stain of the neoplastic cells shows focal reactivity to MDM2.

On macroscopic view, the specimen was a grey‐whitish, solid, soft tissue mass measuring 10 × 5.3 × 3.1 cm. Histopathologically, it revealed a picture of abundant hyperchromatic spindle cells and pleomorphic cells with myxoid background. Immunohistochemical study of the neoplastic cells was positive for vimentin, actin, and MDM2. The morphology and immunohistochemistry (IHC) stain were compatible with the diagnosis of pulmonary artery intimal sarcoma (Fig. 2). However, the surgical margin could not be evaluated because of intraoperative tumour fragmentation.

After surgery, the patient received adjuvant chemotherapy with 6 cycles of combination chemotherapy regimen of epirubicin, ifosfamide, and mesna (MAI) regimen Q3W (epirubicin 60 MG/M2, ifosfamide 1.8 GM/M2, and mesna 0.36 GM/M2) as the chest CT showed residual tumour with direct compression over the graft. A series of subsequent chest CTs showed regression of the residual tumour and releasing of the compression.

## Discussion

Pulmonary artery sarcoma is an extremely rare pulmonary malignancy. It was first described by Mandelstamm in 1923 1. Since then, around 400 cases have been reported 2, 3. The reported mean age is 50 years 4, 5, 6.

Pulmonary artery sarcoma arises from either the intimal wall or intramural wall of the pulmonary artery and can be accordingly classified into intimal and intramural sarcomas 7. In intimal sarcomas as seen in our case, the microscopic examination reveals proliferation of spindle cells in a myxoid background with a high mitotic index and varying degrees of cellular pleomorphisms. It might be admixed with a focal lesion of a more differentiated sarcoma such as leiomyosarcoma, spindle cell sarcoma, rhabdomyosarcoma, chondrosarcoma, osteosarcoma, or angiosarcoma 7, 8. Due to the finding of a more differentiated sarcoma, it has been hypothesized that the intimal sarcoma develops from the pluripotent mesenchymal cells of the intimal artery, which have a potential for multidirectional differentiation 9. Immunohistochemically, vimentin, smooth muscle actin (SMA), desmin, actin, and an endothelial cell marker such as CD31 might be expressed 8, 9. The tumour also express MDM2 3. In our case, the morphological and immunohistochemical stain was compatible with the pattern of a sarcoma.

Pulmonary intimal artery sarcoma occupies the lumen of pulmonary artery and results in right ventricular dysfunction. Symptoms and signs of chronic right ventricular failure are expected. The most common symptoms of pulmonary artery sarcoma are insidious dyspnoea, which is a non‐specific symptom and might be associated with other aetiologies of right ventricular failure. Other symptoms in cases of pulmonary artery sarcoma include tightness in the chest, cough, haemoptysis, and constitutional symptoms. Constitutional symptoms such as fever, fatigue, and weight loss are the features of malignancy. These could help in differentiating pulmonary artery sarcoma 10 from other aetiologies of right ventricular failure. However, physicians seldom establish a diagnosis of pulmonary intimal artery sarcoma based on symptoms alone. Other investigational modalities are usually required. From published studies, the modalities that have been used are echocardiography, CT, magnetic resonance imaging (MRI), and positron emission tomography (PET) 10.

Transthoracic echocardiography provides information about pulmonary artery hypertension, pulmonary valve, and right ventricular function. Besides, when a mass is seen over the pulmonary valve, transthoracic echocardiography can indicate the involvement of the mass and reveal whether the mass moves with the heartbeat. However, detection of the involvement of pulmonary valve and right ventricle with the tumour depends on the operator's experience. In a cohort of nine patients, transthoracic echocardiography provided information about the functioning of the pulmonary valve and right ventricle. However, none of the cases were diagnosed as pulmonary artery sarcoma solely on echocardiography 10. Other radiological modalities are therefore inevitably conducted for confirmation of the diagnosis.

The radiological features of pulmonary artery sarcoma are filling defect in the pulmonary artery lumen, extraluminal tumour invasion, and a nodular appearance. However, despite specific features, published studies have shown that more than half of the patients with pulmonary artery sarcoma are initially misdiagnosed as pulmonary thromboembolism 3, 6, 10. Several studies have been conducted in an attempt to distinguish between the two entities. It has been reported that the CT morphology and location of the lesion help to distinguish between pulmonary artery sarcoma and chronic pulmonary thromboembolism. Pulmonary artery sarcomas are characterized by tumour deposits. Thus, the CT features are characteristic with expansive growth and a bulging appearance against the direction of blood flow, a lobulated structure at the proximal part of the tumours, a grape‐like appearance at the distal end of the tumours, and heterogeneous enhancement 3, 10. Pulmonary artery sarcoma can extend until the bifurcation of the pulmonary arteries, the main pulmonary artery, pulmonary valve, and right ventricular outflow tract 10. In contrast, the proximal end of a chronic pulmonary thromboembolus is a straight and cup‐like structure, caused by blood flow against the surface of blood clots 11. Pulmonary thromboembolism often occurs in a peripheral pulmonary artery. It seldom occurs in the pulmonary trunk or in the pulmonary valve because of faster blood flow in these areas, as mentioned above 11. The filling defect in the pulmonary trunk, pulmonary valve, and the right ventricle are clues to suspect pulmonary artery sarcoma. In our case, the presence of a nodular appearance at the proximal end of the tumour, the heterogeneous contrast enhancement and invasion of the pulmonary trunk aroused the suspicion of a sarcoma and prompted the decision for a surgery.

There are differences between pulmonary artery sarcoma and pulmonary thromboembolism on MRI 11. The contrast MRI picture of a pulmonary artery sarcoma is a non‐homogeneous delayed enhancement; this is not seen in pulmonary thromboembolism. The T2 signal is higher in a pulmonary artery sarcoma than in thromboembolism. However, patients with pulmonary artery sarcoma usually have dyspnoea and are barely able to hold their breath throughout the MRI procedure 3.

18F‐F‐18 fluorodeoxyglucose (FDG) PET/CT is reported to help in differentiating pulmonary artery sarcoma from chronic thromboembolism based on the maximal standardized uptake value (SUVmax). The SUVmax of pulmonary artery sarcoma is significantly higher than that of pulmonary thromboembolism. At the cut‐off value of 3.3, the sensitivity, specificity, and accuracy are reported to be 98.4%, 96.8%, and 97.8%, respectively 12. The common cause for a false negative report is low cellularity of the lesion. Kriz et al. 13 and Lee et al. 14 reported cases with poor uptake of FDG in PET in patients with pulmonary intimal sarcoma. The histopathology of these cases showed highly malignant cells but with low cellularity and significant interstitial myxoid tissue type. Finally, PET had a benefit in establishing the pulmonary artery sarcoma staging.

The published treatment modalities include resection surgery, targeted therapy, radiotherapy, and chemotherapy. Complete surgical excision with pulmonary endarterectomy is recommended as it provides the best survival benefit 4, 5. According to recent studies, multimodal treatment with adjuvant or neoadjuvant chemotherapy and/or radiotherapy might result in a favourable survival curve compared to monotherapy such as surgery alone 4, 6, 15, 16. However, further studies are required to confirm the benefit. Chemotherapy regimens include adriamycin plus isosfamide, gemcitabine plus taxane, or dacerbazine 4. As for targeted therapy, a multi‐targeted receptor tyrosine kinase inhibitor, panzopanib, which was approved for advanced soft tissue sarcoma by the Food and Drug Administration (FDA), has been reported to be useful in treating pulmonary artery sarcoma. Kollár et al. 17 and Funatsu et al. 18 described a partial response of pulmonary artery sarcoma to panzopanib. Combination of radiotherapy and panzopanib as a second‐line treatment was also described 15. However, owing to the limited available evidence, their positive response over panzopanib alone warrants further evaluation.

In our case, there was residual tumour after the surgery, causing external compression. After adjuvant chemotherapy with epirubicin plus isosfamide, regression of the residual tumour was observed and there was release of external compression.

The prognosis of pulmonary artery sarcoma is very poor. The survival without treatment is only 1.5 months 5. A study 4 reported that the median survival was prolonged to 36.5 ± 20.2 months with complete surgical resection versus 11 ± 3 months with incomplete surgical resection. The study also reported that the median survival was 24 ± 8 months for those with multimodal treatment compared to eight months with monotherapy.

### Disclosure Statement

Appropriate written informed consent was obtained for publication of this case report and accompanying images.

## References

[1] Mandelstamm M . 1923 Über primäre Neubildungen des Herzens. Virchows. Arch. Pathol. Anat. 245:43–54.

[2] Mussot S , Ghigna M‐R , Mercier O , et al. 2013 Retrospective institutional study of 31 patients treated for pulmonary artery sarcoma. Eur. J. Cardiothorac. Surg. 43:787–793. 22843511 10.1093/ejcts/ezs387

[3] Moguillansky MI , Verma N , Shah P , et al. 2019 Pulmonary artery sarcoma: case report and review of the literature. Respir. Med. Case Rep. 27:100857. 10.1016/j.rmcr.2019.100857 PMC6538954 31193694

[4] Blackmon SH , Rice DC , Correa AM , et al. 2009 Management of primary pulmonary artery sarcomas. Ann. Thorac. Surg. 87(3):977–984. 19231448 10.1016/j.athoracsur.2008.08.018

[5] Deng L , Zhu J , Xu J , et al. 2018 Clinical presentation and surgical treatment of primary pulmonary artery sarcoma. Interact. Cardiovasc. Thorac. Surg. 26(2):243–247. 29049745 10.1093/icvts/ivx318

[6] Lee Y , Kim HJ , Yoon H , et al. 2016 Clinical characteristics and treatment outcomes of primary pulmonary artery sarcoma in Korea. J. Korean Med. Sci. 31(11):1755–1760. 27709853 10.3346/jkms.2016.31.11.1755 PMC5056207

[7] Cox J , Chiles C , Aquino SL , et al. 1997 Pulmonary artery sarcomas: a review of clinical and radiologic features. J. Comput. Assist. Tomogr. 21:750–755. 9294569 10.1097/00004728-199709000-00018

[8] Keel SB , Bacha E , Mark E , et al. 1999 Primary pulmonary sarcoma: a clinicopathologic study of 26 cases. Mod. Pathol. 12:1124–1131. 10619264

[9] Chen D , Zhu G , Wang D , et al. 2016 Clinicopathological and immunohistochemical features of pulmonary artery sarcoma: a report of three cases and review of the literature. Oncol. Lett. 11(4):2820–2826. 27073558 10.3892/ol.2016.4308 PMC4812211

[10] Pu X , Song M , Huang X , et al. 2018 Clinical and radiological features of pulmonary artery sarcoma: a report of nine cases. Clin. Respir. J. 12(5):1820–1829. 29115002 10.1111/crj.12743

[11] Kim C , Kim MY , Kang J‐W , et al. 2018 Differential diagnosis of pulmonary sarcoma and CPTPH using CT and MRI. Korean J. Radiol. 19(4):792–802. 29962886 10.3348/kjr.2018.19.4.792 PMC6005959

[12] Xi XY , Gao W , Gong JN , et al. 2019 Value of 18F‐FDG PET/CT in differentiating malignancy of pulmonary artery from pulmonary thromboembolism: a cohort study and literature review. Int. J. Cardiovasc. Imaging 35(7):1395–1403. 30747369 10.1007/s10554-019-01553-5

[13] Kriz JP , Munfakh NA , King GS , et al. 2016 Intimal sarcoma of the pulmonary artery with unusual findings: a case report. Case Rep. Oncol. 9(1):267–272. 27239183 10.1159/000445498 PMC4881246

[14] Lee DH , Jung TE , Lee JH , et al. 2013 Pulmonary artery intimal sarcoma: poor 18F‐fluorodeoxyglucose uptake in positron emission computed tomography. J. Cardiothorac. Surg. 8:40. 23497592 10.1186/1749-8090-8-40 PMC3605187

[15] Chiola I , Belgioia L , Vaccara EML , et al. 2019 Reirradiation of pulmonary artery intimal sarcoma: a case report. Clin. Case Rep. 7(7):1342–1346. 31360482 10.1002/ccr3.2216 PMC6637327

[16] Secondino S , Grazioli V , Valentino F , et al. 2017 Multimodal approach of pulmonary artery intimal sarcoma: a single‐institution experience. Sarcoma 2017:7941432. 10.1155/2017/7941432 PMC5585613 28912665

[17] Kollár A , Jones RL , Stacchiotti S , et al. 2017 Azopanib in advanced vascular sarcomas: an EORTC Soft Tissue and Bone Sarcoma Group (STBSG) retrospective analysis. Acta Oncol. 56(1):88–92. 27838944 10.1080/0284186X.2016.1234068

[18] Funatsu Y , Hirayama M , Shiraishi J , et al. 2016 Intimal sarcoma of the pulmonary artery treated with pazopanib. Intern. Med. 55(16):2197–2202. 27522994 10.2169/internalmedicine.55.6199

